# Aripiprazole-induced rosacea. Case report and literature review

**DOI:** 10.1192/j.eurpsy.2021.1295

**Published:** 2021-08-13

**Authors:** T. Gutiérrez Higueras, B. Hernandez Gajate, F. Calera Cortés, S. Vicent Forés, S. Sainz De La Cuesta Alonso

**Affiliations:** Clinical Unit Of Mental Health., Reina Sofia University Hospital., Córdoba., Spain

**Keywords:** aripiprazole, skin reaction, adverse effect, antipsychotic

## Abstract

**Introduction:**

Skin and subcutaneus tissue disorders are common type of adverse drug reactions reported with a wide variety of both typical and atypical antipsychotics. Aripiprazole is a quinolinone antipsychotic that is a partial agonist at the D2 and 5-HT1A receptors and antagonist at the 5-HT2A receptors. We report a case of rosacea that developed after starting aripiprazole in a patient with schizophrenia and which remitted after the drug was stopped.

**Objectives:**

To present a case of rosacea that developed in a schizophrenic patient after starting aripiprazole. Review of literature and search for the total number of cases reported in the european database of suspected adverse drug reactions (EudraVigilance).

**Methods:**

We carried out a literature review in Pubmed electing those articles focused on skin ad subcutaneous skin disorders in those patients that have been taking aripiprazole. Review number of cases of skin reactions reported by the European database of suspected adverse drug reactions.

**Results:**

A 43-year-old man previously diagnosed with schizophrenia with low adherence to different treatments. He came to our service seeking for help in order to decrease delusions with a treatment with minimun adverse reactions. We started aripirazole 10 mg every day and, after 7 days appeared signs of rosacea in his face. After discontinuation of aripiprazol, after 5 days, rosacea remitted.

**Conclusions:**

Rosacea in our case posibly point aripiprazole as the agent that produced the skin reaction. After stopping the treatment the signs disapeared. Awareness of skins manifestations produced by aripiprazole is essential to prevents worse skin reactions.

